# The complete chloroplast genome sequence of *Bambusa stenoaurita* (Bambusoideae)

**DOI:** 10.1080/23802359.2021.1944373

**Published:** 2021-07-14

**Authors:** Haitao Xia, Xing Liu, Yueying Wang, Xiaowen Li, Jinwang Wang, Chuan Jin

**Affiliations:** Zhejiang Institute of Subtropical Crops, Wenzhou, Zhejiang, PR China

**Keywords:** *Bambusa stenoaurita*, chloroplast genome, phylogeny, Bambusoideae

## Abstract

*Bambusa stenoaurita* is an excellent sympodial bamboo species, which is cultivated for its shoots in some parts of China. Here, we sequenced and reported the complete chloroplast genome of *B. stenoaurita* for the first time. The complete chloroplast genome sequence of *B. stenoaurita* was generated by *de novo* assembly using whole-genome next-generation sequencing. The genome was 139,451 bp in total length, including a large single-copy region of 82,958 bp, a small single-copy region of 12,897 bp, a pair of invert repeats regions of 21,798 bp. The plastid genome contained 134 genes including 87 protein-coding genes, 39 tRNA genes, and 8 rRNA genes. Phylogenetic analysis based on 23 chloroplast genomes demonstrates that *B. stenoaurita* is closely related to *B. emeiensis* in Bambusoideae.

*Bambusa stenoaurita* (W.T. Lin) T.H. Wen (http://www.theplantlist.org) is known for a large-scale sympodial bamboo species. Its shoots are delicious and are considered a high-quality vegetable in China. Wenzhou is located on the northern edge of the south subtropical zone. In 2014, *B. stenoaurita* was first introduced in Hua'an, Fujian province, and exhibited excellent ecological adaptability, promising growth, and consumption potential. The chloroplasts (cp) genome, which has a maternal inheritance and a conserved structure, has been used to investigate plant development and phylogenetic analysis (Wang et al. 2018). In this study, we characterized the complete cp genome sequence of *B. stenoaurita* using Illumina sequencing data, to understand the phylogenetic position of the species for future evolutionary studies.

*B. stenoaurita* leaves specimens were sampled from Wenzhou city, Zhejiang Province, China (N 28°0′6″, E 120°37′53″), and immediately dried with silica gel for DNA extraction. DNA was sent to Wuhan Bena Biotechnology Co., Ltd. to construct DNA library and sequenced by Illumina HiSeq 6000 sequencing platform (Illumina, San Diego, CA). The Specimens have been stored in the Herbarium of the Zhejiang Institute of Subtropical Crops (Resource person: Haitao Xia; Email: summerxht@hotmail.com) the specimen code BS. In addition, the Illumina High-throughput sequencing platform data were filtered by the script in the NOVOPlasty (Dierckxsens et al. 2017). After the DNA extraction from fresh leaf tissues, its quantification was validated using Agarose gel electrophoresis and Nanodrop concentration 500 bp randomly interrupted by the Covaris ultrasonic breaker for library construction. Approximately, 2.0 GB of raw data were generated with 150 bp paired-end read lengths. The cp genome of *B. stenoaurita* was assembled by GetOrganelle (https://github.com/Kinggerm/GetOrganelle) using the cp genome of *Phyllostachys edulis* (GeneBank accession: HQ337796) as a reference, it can get the plastid-like reads, and the reads were viewed and edited by Bandage (Wick et al. 2015). The cp genome annotation was assembled based on the comparison by Geneious v 11.1.5 (Biomatters Ltd, Auckland, New Zealand) (Kearse et al. 2012).

The final complete cp genome sequence of *B. stenoaurita* has been submitted to GenBank with the accession number MW540515. Raw reads were deposited in the GenBank Sequence Read Archive (SRA PRJNA695357). The complete cp genome of *B. stenoaurita* was a circular shape of 139,451 bp in length, consisting of four distinct regions, such as a large single-copy (LSC) region of 82,958 bp, a small single-copy (SSC) region of 12,897 bp, and a pair of inverted repeats (IR) regions of 21,798 bp. The complete cp genome consisted of 134 genes, including 87 protein-coding genes, 39 tRNA genes, and 8 rRNA genes. The overall GC content of the chloroplast genome was 38.91%, and the corresponding GC values in LSC, SSC, and IR regions were 37.00%, 33.23%, and 44.21%, respectively. Phylogenetic analyses including *B. stenoaurita*, 21 other Bambusoideae species, and two outgroups of Pooideae, Panicoideae were performed using complete cp genomes. All of them were downloaded from NCBI GenBank. The full-length sequences were aligned by MAFFT v7.307 software (Katoh and Standley 2013), and the phylogenetic tree was constructed by RAxML (Stamatakis 2014) with 1000 bootstrap replicates. The GTRGAMMA model was used in the ML analysis. The phylogenetic tree reveals that *B. stenoaurita* is closely related to *B. emeiensis* with strong support as shown in Figure 1.

**Figure 1.**
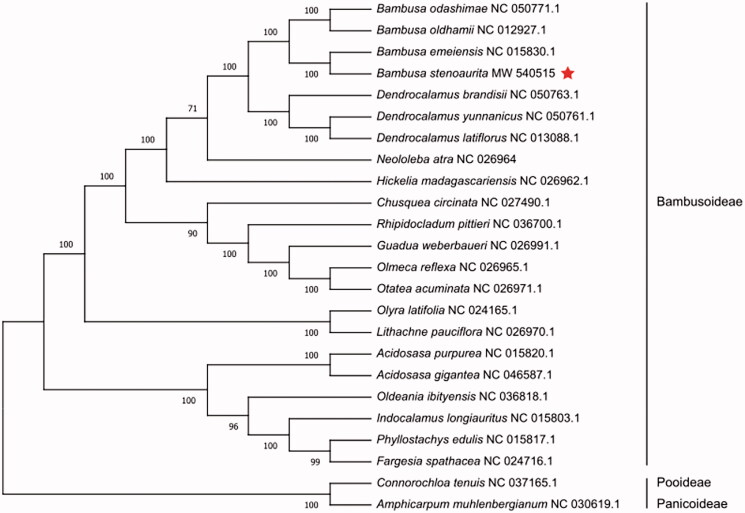
Maximum-likelihood phylogenetic tree based on complete cp genomes. Numbers close to each node are bootstrap support values.

## Data Availability

The genome sequence data that support the findings of this study are openly available in GeneBank at of NCBI at (https://www.ncbi.nlm.nih.gov/nuccore/ MW540515) under the accession no. MW540515. The associated BioProject, SRA, and Bio-Sample numbers are PRJNA695357, SRX9966999, and SAMN17612304, respectively.
